# Prevalence and trends of autism spectrum disorder and other developmental disabilities among children and adolescents in the United States from 2019 to 2021

**DOI:** 10.3389/fpsyt.2024.1471969

**Published:** 2024-09-26

**Authors:** Wenrong Ge, Cancan Zhang, Guang Yang, Bo Zhang

**Affiliations:** ^1^ Department of Pediatrics, Beijing Friendship Hospital, Capital Medical University, Beijing, China; ^2^ Division of General Medicine, Beth Israel Deaconess Medical Center, Harvard Medical School, Boston, MA, United States; ^3^ Department of Pediatrics, the First Medical Center of Chinese PLA General Hospital, Beijing, China; ^4^ Senior Department of Pediatrics, the Seventh Medical Center of Chinese PLA General Hospital, Beijing, China; ^5^ Medical School of Chinese PLA, Beijing, China; ^6^ Department of Neurology, Boston Children’s Hospital, Harvard Medical School, Boston, MA, United States; ^7^ Biostatistics and Research Design Center, Institutional Centers for Clinical and Translational Research, Boston Children’s Hospital, Harvard Medical School, Boston, MA, United States

**Keywords:** neurodevelopmental disorders, learning disability, National Health Interview Survey (NHIS) data, racial disparities, prevalence of autism spectrum disorder

## Abstract

**Background:**

The National Health Interview Survey (NHIS) is a comprehensive health survey conducted by the National Center for Health Statistics (NCHS) in the U.S., providing valuable insights into the health status of the population. This study focuses on the NHIS child survey between 2019 and 2021, exploring developmental disabilities in U.S. children, including autism spectrum disorder (ASD), attention deficit/hyperactivity disorder or attention-deficit disorder (ADHD/ADD), intellectual disability (ID), other developmental delay (other DD), and learning disability (LD).

**Objective:**

Leveraging NHIS data, our primary objective is to investigate the latest trends and disparities in the prevalence of developmental disabilities among various racial-ethnic groups.

**Methods:**

Employing a repeated cross-sectional design, we analyzed NHIS data from 2019 to 2021, focusing on children aged 3-17. The survey employed a meticulous stratified multi-stage sampling design. We utilized SAS version 9.4 for data analysis, calculating race-ethnicity-specific prevalence rates and employing weighted linear regression and the Rao-Scott chi-square test for trend analysis.

**Results:**

Among 19,490 children, prevalence rates varied: ASD (3.11%), ADHD/ADD (9.50%), ID (1.85%), other DD (5.66%), and LD (7.49%). Non-Hispanic black children exhibited higher rates of ID and LD, while non-Hispanic white children had the highest ADHD/ADD prevalence. Disparities persisted across sociodemographic subgroups, with variations in prevalence rates.

**Conclusion:**

Our study reveals an increase in ASD prevalence and persistent disparities among racial-ethnic groups. Non-Hispanic black children face elevated risks of ID and LD, while non-Hispanic white children exhibit higher rates of ADHD/ADD.

## 1 Introduction

The National Health Interview Survey (NHIS) is an extensive and ongoing survey conducted by the National Center for Health Statistics (NCHS), a part of the Centers for Disease Control and Prevention (CDC) in the United States (U.S.) (1, 2). The primary objective of the NHIS is to gather data on a broad spectrum of health-related topics, encompassing health status, healthcare access, health behaviors, and healthcare usage. It yields valuable insights into health trends and the challenges faced by the American population. The NHIS comprises both adult and child surveys and stands as one of the most vital sources of information regarding the health and well-being of the U.S. populace (2). The child survey within the NHIS between 2019 and 2021 encompassed various sections dedicated to developmental disabilities in U.S. children and adolescents. It addressed conditions including autism spectrum disorder (ASD), attention deficit/hyperactivity disorder or attention-deficit disorder (ADHD/ADD), intellectual disability (ID), any other developmental delay (other DD), and learning disability (LD) (3). These conditions typically manifest early in the developmental stages and can lead to personal, social, learning, language, and behavioral challenges.

Previous studies by Boyles et al. (4) and Zaboltsky et al. (5) investigated the prevalence and trends of these developmental disabilities among U.S. children and adolescents, spanning from 1997 to 2008 and from 2009 to 2017, respectively. Both studies reported an increase in prevalence during their respective periods. Additionally, Kogan et al. (6) and Xu et al. (7) conducted an analysis of the 2016 National Survey of Children’s Health (NSCH), concluding that the prevalence of ASD in the U.S. in 2016 was 2.50%, indicating 1 in 40 U.S. children was autistic in 2016. Magaña et al. (8) utilized data from the 2005-2006 wave of the National Survey of Children with Special Health Care Needs to investigate racial-ethnic disparities in the quality of healthcare among children with ASD, Down syndrome, cerebral palsy, and Intellectual Disability. They compared black and Latino children with white children and identified racial-ethnic disparities in five out of six healthcare quality outcomes (8). Magaña et al. repeated this investigation for the 2009-2010 wave of the survey and found that these disparities remained unchanged (9).

This study leveraged data released by the NHIS from 2019 to 2021. Our primary objective was to investigate the latest trends and disparities in the prevalence of developmental disabilities among various racial-ethnic groups.

## 2 Methods

This study adopted a repeated cross-sectional design by consolidating data gathered through the NHIS surveys conducted between 2019 and 2021. The NHIS employed a meticulously structured stratified multi-stage sampling design and conducted interviews with either adults or parents and guardians of children, with the aim of collecting comprehensive information regarding the health conditions of U.S. residents (10, 11). The NHIS’s sampling plan was thoughtfully crafted by the Division of Health Interview Statistics within the National Center for Health Statistics at the Centers for Disease Control and Prevention. The NHIS waves from 2019 to 2021 and from previous years were conducted independently from one another, following the stratified multi-stage sampling design each year. As a result, the data collected from these individual years can be combined for analysis while maintaining the independence of each wave. For a more in-depth understanding of the sampling design and its implementation, the Survey Design document of the NHIS provides detailed information (10).

In our study, we focused on children and adolescents between the ages of 3 and 17, drawing data from the 2019, 2020, and 2021 waves of the NHIS. Our analysis revolved around the responses to the NHIS survey questions related to developmental disabilities. These questions are as follows. Parents or guardians were asked, “Does [sample child’s name] currently have Autism, Asperger’s disorder, pervasive developmental disorder, or autism spectrum disorder?” for ASD. They were also asked, “Does [sample child’s name] currently have Attention-Deficit/Hyperactivity Disorder or ADHD, or Attention-Deficit Disorder or ADD?” for ADHD/ADD. For ID, the question was, “Does [sample child’s name] currently have an intellectual disability, also known as Intellectual Disability?” Additionally, there was a subsequent question, “Does [sample child’s name] still have any other developmental delay?” for other DD. Lastly, the survey included a question, “Does [sample child’s name] currently have a learning disability?” to assess LD. Our analysis sought to examine the outcomes of these survey questions in conjunction with sociodemographic characteristics. Furthermore, our objective was to investigate the most recent trends and disparities in the prevalence of these developmental disabilities among various racial-ethnic groups. The dataset for our study also encompassed sociodemographic characteristics of the sample children, including age, sex, racial-ethnic groups (non-Hispanic white, non-Hispanic black, Hispanic, non-Hispanic others; these categories were designed by the NHIS survey and adopted here), the highest level of education attained by all adults in the sample children’s family, and the income-to-poverty ratio of the sample children’s family.

Following NHIS guidelines (10, 11), we executed the survey procedure using SAS version 9.4 (SAS Institute Inc.) to calculate race-ethnicity-specific prevalence rates of developmental disabilities, along with 95% confidence intervals (CIs). We provided stratified estimates considering age, gender, highest family education level, and family income-to-poverty ratio. To assess the association between race/ethnicity and prevalence, we conducted the Rao-Scott chi-square test to examine independence. Additionally, we employed weighted linear regression analysis to identify trends, with statistical significance defined as a p-value less than 0.05. Our study did not involve research on human subjects and animals, and thus, it was exempt from the requirement for ethical approval.

## 3 Results

A total of 19,490 children and adolescents, aged 3 to 17 years, participated in the NHIS survey conducted between 2019 and 2021. Among the participants across the three years, the results indicated the following prevalence rates: 3.11% (CI: [2.80%, 3.41%]) reported being diagnosed with ASD; 9.50% (8.95%, 10.04%) had ADHD/ADD; 1.85% (1.61%, 2.09%) had ID; 5.66% (5.23%, 6.08%) had other DD; 7.49% (6.99%, 7.99%) had LD (**Table 1**, top section). Non-Hispanic black children exhibited the highest prevalence rates of ID (2.82%, CI: [1.84%, 3.80%]) and LD (9.40%, CI: [7.67%, 11.13%]) in comparison to other racial/ethnic groups (p=0.0117 and 0.0006, respectively). Non-Hispanic white children had the highest prevalence of ADHD/ADD (11.18%, CI: [10.41%, 11.95%], p<0.0001). No significant differences were observed in the prevalence of ASD and other DD among various racial-ethnic groups (**Table 1**, top section).

**Table 1:** Weighted prevalence of autism spectrum disorder (ASD), attention-deficit/hyperactivity disorder or attention deficit disorder (ADHD/ADD), intellectual disability (ID), any other developmental disabilities (Other DD), and learning disabilities (LD) in the US children and adolescents aged 3 to 17 by race/ethnicity in the National Health Interview Survey, 2019-2021.

Characteristics		Weighted prevalence, % (95% confidence interval)					P-value^b^
		All	Non-Hispanic White	Non-Hispanic Black	Hispanic	Non-Hispanic Others	
ASD		3.11 (2.80,3.41)	3.06 (2.68,3.44)	3.56 (2.41,4.71)	2.96 (2.38,3.54)	3.15 (2.3,4.01)	0.7262
ADHD/ADD		9.50 (8.95,10.04)	11.18 (10.41,11.95)	9.64 (7.98,11.31)	7.32 (6.41,8.23)	6.26 (5.06,7.45)	<0.0001
ID		1.85 (1.61,2.09)	1.75 (1.44,2.07)	2.82 (1.84,3.80)	1.77 (1.32,2.23)	1.29 (0.81,1.77)	0.0117
Other DD		5.66 (5.23,6.08)	5.99 (5.41,6.56)	6.42 (4.91,7.93)	5.13 (4.29,5.97)	4.36 (3.38,5.33)	0.0526
LD		7.49 (6.99,7.99)	7.60 (6.90,8.29)	9.40 (7.67,11.13)	7.24 (6.35,8.13)	5.17 (4.11,6.24)	0.0006
ASD	Age						
	3-11	3.08 (2.68,3.48)	2.81 (2.31,3.31)	3.83 (2.22,5.45)	3.11 (2.36,3.86)	3.38 (2.26,4.50)	0.4490
	12-17	3.14 (2.66,3.63)	3.40 (2.76,4.04)	3.19 (1.80,4.58)	2.73 (1.83,3.63)	2.79 (1.55,4.02)	0.6416
	Sex						
	Male	4.65 (4.15,5.15)	4.65 (4.01,5.29)	5.19 (3.34,7.04)	4.35 (3.40,5.31)	4.75 (3.34,6.16)	0.8318
	Female	1.49 (1.18,1.81)	1.42 (1.05,1.80)	1.81 (0.72,2.89)	1.48 (0.79,2.16)	1.50 (0.65,2.36)	0.8998
	Education^c^						
	High School	2.74 (2.15,3.33)	2.83 (1.92,3.75)	3.55 (1.70,5.41)	2.48 (1.55,3.41)	2.16 (0.40,3.91)	0.6063
	College	3.45 (3.03,3.86)	3.50 (2.96,4.05)	3.31 (1.96,4.67)	3.35 (2.51,4.19)	3.55 (2.28,4.81)	0.9852
	Graduate	2.68 (2.05,3.32)	2.34 (1.69,2.99)	4.45 (0.32,8.58)	3.09 (1.24,4.94)	3.02 (1.64,4.40)	0.3641
	Income Ratio^d^						
	<1	4.61(3.55,5.68)	5.25(3.38,7.13)	5.04(2.31,7.77)	4.32(2.66,5.97)	2.74(0.57,4.91)	0.6127
	1-2	2.91(2.26,3.56)	4.05(2.97,5.13)	2.50(0.49,4.51)	1.81(1.11,2.51)	3.58(1.62,5.54)	0.0293
	2-4	3.10(2.59,3.62)	3.04(2.38,3.70)	3.24(1.47,5.00)	2.8(1.92,3.68)	4.07(2.13,6.01)	0.6428
	>4	2.46(2.02,2.89)	2.23(1.74,2.71)	3.39(0.17,6.60)	3.52(2.07,4.97)	2.30(1.34,3.25)	0.2908
ADHD/ADD	Age						
	3-11	6.81 (6.17,7.46)	7.92 (6.97,8.87)	6.97 (5.05,8.88)	5.29 (4.25,6.33)	5.04 (3.61,6.47)	0.0007
	12-17	13.31 (12.41,14.22)	15.68 (14.38,16.99)	13.26 (10.45,16.06)	10.30 (8.64,11.96)	8.21 (6.17,10.26)	<0.0001
	Sex						
	Male	12.26 (11.48,13.04)	14.29 (13.2,15.37)	12.76 (10.12,15.39)	9.75 (8.31,11.18)	7.76 (6.00,9.53)	<0.0001
	Female	6.61 (5.98,7.25)	7.98 (7.04,8.93)	6.29 (4.32,8.27)	4.74 (3.67,5.82)	4.70 (3.16,6.23)	<0.0001
	Education^c^						
	High School	9.93 (8.79,11.07)	14.36 (12.14,16.58)	11.55 (7.91,15.2)	6.90 (5.54,8.27)	6.80 (3.43,10.16)	<0.0001
	College	10.09 (9.34,10.85)	11.72 (10.64,12.79)	9.94 (7.83,12.04)	7.72 (6.44,9.01)	7.03 (5.35,8.71)	<0.0001
	Graduate	7.67 (6.76,8.57)	8.72 (7.48,9.97)	4.61 (2.40,6.82)	7.11 (4.76,9.47)	4.57 (2.99,6.16)	0.0009
	Income Ratio^d^						
	<1	12.67(11.03,14.31)	19.03(15.58,22.47)	13.63(9.75,17.51)	8.56(6.45,10.66)	8.36(4.30,12.42)	<.0001
	1-2	10.27(9.15,11.40)	15.30(13.14,17.45)	9.21(6.46,11.96)	6.17(4.79,7.54)	8.97(5.94,11.99)	<.0001
	2-4	8.16(7.34,8.97)	9.50(8.33,10.67)	6.50(4.46,8.55)	6.94(5.22,8.67)	5.20(3.24,7.16)	0.0020
	>4	8.53(7.72,9.34)	9.22(8.22,10.22)	8.05(4.41,11.70)	8.49(6.17,10.81)	4.54(3.12,5.96)	0.0036
ID	Age						
	3-11	1.59 (1.28,1.91)	1.64 (1.21,2.06)	2.09 (0.80,3.37)	1.37 (0.80,1.93)	1.37 (0.74,1.99)	0.5741
	12-17	2.21 (1.82,2.59)	1.91 (1.45,2.38)	3.81 (2.21,5.41)	2.37 (1.58,3.16)	1.17 (0.35,2.00)	0.0053
	Sex						
	Male	2.31 (1.93,2.69)	2.24 (1.74,2.73)	3.01 (1.54,4.48)	2.37 (1.59,3.15)	1.60 (0.84,2.36)	0.3788
	Female	1.37 (1.08,1.65)	1.26 (0.90,1.62)	2.62 (1.33,3.91)	1.14 (0.66,1.62)	0.97 (0.36,1.58)	0.0078
	Education^c^						
	High School	2.30 (1.76,2.84)	2.84 (1.78,3.90)	2.57 (1.11,4.03)	1.91 (1.17,2.64)	1.83 (0.24,3.42)	0.4525
	College	1.78 (1.47,2.08)	1.74 (1.33,2.15)	2.30 (1.30,3.29)	1.82 (1.15,2.50)	1.17 (0.50,1.85)	0.3792
	Graduate	1.55 (1.04,2.06)	1.27 (0.81,1.72)	5.21 (0.59,9.84)	0.95 (0.12,1.77)	1.19 (0.43,1.96)	0.0001
	Income Ratio^d^						
	<1	2.53(1.8,3.27)	2.96(1.59,4.32)	2.81(1.06,4.56)	2.17(1.04,3.30)	2.10(0.25,3.95)	0.7849
	1-2	2.30(1.70,2.91)	2.26(1.36,3.16)	3.13(0.88,5.39)	2.13(1.19,3.06)	1.71(0.47,2.95)	0.6417
	2-4	1.43(1.09,1.77)	1.58(1.08,2.07)	2.25(0.82,3.67)	0.98(0.48,1.48)	0.71(0.03,1.40)	0.0908
	>4	1.54(1.19,1.90)	1.44(1.02,1.85)	3.25(0.79,5.71)	1.74(0.77,2.70)	1.17(0.47,1.88)	0.0946
Other DD	Age						
	3-11	6.19 (5.57,6.81)	6.77 (5.94,7.60)	7.12 (5.04,9.20)	5.35 (4.03,6.67)	4.35 (3.16,5.53)	0.0390
	12-17	4.90 (4.33,5.47)	4.90 (4.16,5.64)	5.48 (3.43,7.53)	4.80 (3.63,5.97)	4.37 (2.93,5.81)	0.8450
	Sex						
	Male	7.29 (6.59,7.99)	7.53 (6.65,8.40)	8.36 (6.09,10.62)	6.94 (5.45,8.42)	5.63 (4.13,7.14)	0.2552
	Female	3.93 (3.45,4.42)	4.37 (3.68,5.05)	4.34 (2.59,6.09)	3.21 (2.33,4.09)	3.03 (1.87,4.20)	0.1599
	Education^c^						
	High School	5.29 (4.45,6.14)	5.83 (4.42,7.25)	6.20 (3.57,8.83)	4.58 (3.38,5.79)	5.43 (2.45,8.41)	0.4974
	College	5.72 (5.14,6.29)	5.90 (5.13,6.67)	6.31 (4.38,8.24)	5.43 (4.27,6.59)	4.62 (3.30,5.94)	0.4865
	Graduate	5.90 (5.01,6.79)	6.22 (5.15,7.29)	7.28 (2.67,11.89)	5.97 (3.41,8.53)	3.28 (2.07,4.48)	0.1527
	Income Ratio^d^						
	<1	6.37(5.14,7.60)	7.10(5.17,9.03)	6.88(3.76,10.00)	5.65(3.67,7.63)	5.98(2.87,9.09)	0.7542
	1-2	6.14(5.19,7.08)	7.33(5.68,8.99)	8.08(5.01,11.15)	4.66(3.46,5.87)	3.87(1.96,5.77)	0.0085
	2-4	5.27(4.58,5.96)	5.61(4.69,6.53)	5.32(2.55,8.10)	5.04(3.58,6.50)	3.75(2.00,5.51)	0.5816
	>4	5.3(4.59,6.01)	5.52(4.67,6.37)	3.72(1.46,5.98)	5.45(3.56,7.33)	4.51(2.91,6.11)	0.4808
LD	Age						
	3-11	6.23 (5.63,6.84)	6.36 (5.49,7.24)	8.14 (5.96,10.32)	5.77 (4.76,6.77)	4.50 (3.21,5.80)	0.0228
	12-17	9.28 (8.48,10.08)	9.30 (8.19,10.42)	11.10 (8.53,13.67)	9.40 (7.71,11.09)	6.25 (4.44,8.07)	0.0500
	Sex						
	Male	9.18 (8.43,9.94)	9.20 (8.14,10.25)	11.59 (8.99,14.2)	8.67 (7.27,10.07)	7.33 (5.64,9.02)	0.0382
	Female	5.72 (5.12,6.33)	5.95 (5.08,6.81)	7.04 (5.02,9.06)	5.72 (4.60,6.84)	2.94 (1.82,4.07)	0.0078
	Education^c^						
	High School	9.32 (8.16,10.48)	12.30 (10.12,14.47)	10.88 (7.66,14.09)	7.41 (5.92,8.90)	5.37 (2.64,8.09)	0.0002
	College	7.59 (6.94,8.24)	7.59 (6.72,8.45)	9.02 (6.83,11.20)	7.54 (6.18,8.89)	5.88 (4.26,7.50)	0.1693
	Graduate	5.38 (4.51,6.26)	5.46 (4.44,6.48)	7.67 (2.91,12.43)	5.06 (2.94,7.19)	3.81 (2.24,5.38)	0.2680
	Income Ratio^d^						
	<1	12.16(10.59,13.73)	15.14(12.05,18.23)	13.68(9.77,17.58)	9.94(7.69,12.18)	8.70(4.96,12.44)	0.0185
	1-2	8.30(7.17,9.43)	10.83(8.69,12.98)	8.97(5.83,12.11)	6.06(4.69,7.42)	6.19(3.57,8.81)	0.0011
	2-4	6.15(5.41,6.88)	6.33(5.31,7.34)	7.00(4.07,9.93)	6.19(4.71,7.67)	4.00(2.35,5.65)	0.3212
	>4	5.72(5.01,6.43)	5.73(4.86,6.60)	5.66(2.52,8.80)	7.20(5.02,9.38)	4.07(2.60,5.54)	0.1838

a Weighted point estimates were estimated using SAS version 9.4 survey procedures (SAS Institute Inc).

b P-values were estimated for the difference in prevalence by race/ethnicity groups using Rao-Scott chi-square test.

c Highest level of education of all the adults in sample children’s family.

d Income to poverty ratio.

When we examined the prevalence of ASD among various sociodemographic subgroups, we observed that, for these subgroups, there was generally no significant association between ASD and race/ethnicity (**Table 1**, ASD section). However, a further analysis of our data revealed noteworthy disparities in the prevalence of ADHD/ADD across different racial-ethnic groups within all sociodemographic subgroups. Specifically, among children who were non-Hispanic white and black, we found significantly higher prevalence rates compared to Hispanic children and non-Hispanic others. These differences were consistent across all four sociodemographic factors, with a p-value ranged from less than 0.0001 to 0.0036 (**Table 1**, ADHD/ADD section). These disparities remained in prevalence of LD across different racial-ethnic groups within most sociodemographic subgroups, except four subgroups in highest family education level and income-to-poverty ratio (**Table 1**, LD section). Furthermore, we discovered that non-Hispanic black children aged 12-17 had a significantly higher prevalence rate (p=0.0053) of ID compared to other racial-ethnic groups. Among female children (p=0.0078) and among those whose highest family education level was graduate school (p=0.0001), the non-Hispanic black subgroup exhibited significantly higher prevalence rates of ID (**Table 1**, ID section). Non-Hispanic black children aged 3-11 and whose highest family education level was graduate school (p=0.0001) had a significantly higher prevalence rate (p=0.0390) of other DD compared to other racial-ethnic groups (**Table 1**, other DD section).

## 4 Discussion

Our study provides the latest update on prevalence rates and racial-ethnic disparities for various developmental disabilities, offering a valuable comparison to earlier findings. Yuan, Li, and Lu (12) conducted an analysis of the 2014-2018 waves of the NHIS, reporting an overall ASD prevalence of 2.49%. In a similar vein, Xu et al. (7) examined the prevalence of ASD in the U.S. in 2016, reporting a rate of 2.50% based on their analysis of the NSCH data. However, our analysis revealed a noteworthy change in the prevalence rate of ASD from 2019 to 2021. During this period, the prevalence of ASD in the U.S. increased to 3.11%, signifying a notable rise in ASD prevalence within the country.

Our study also revealed variations in the prevalence of ADHD/ADD, ID, other DD, and LD among different racial-ethnic groups. Notably, non-Hispanic black children exhibited the highest prevalence of ID and LD, while non-Hispanic white children had the highest prevalence of ADHD/ADD. While we observed significant disparities in the prevalence of ADHD/ADD and LD among different racial-ethnic groups, such disparities were not evident in the case of ASD. This suggested a more consistent prevalence of ASD across diverse racial-ethnic backgrounds as reported by Yuan, Li, and Lu (12). Past research has indicated that learning difficulties can be linked to parental involvement in children’s education (13). When parents have lower levels of education or income, their involvement in their children’s education may be reduced, potentially exacerbating disparities in LD (14).

The increase in the prevalence of autism in this study may be due to a variety of factors. One key factor is the improvement in diagnostic criteria and greater awareness among healthcare providers and the public. The definitions of ASD have broadened over time, encompassing a wider range of behaviors and developmental issues, leading to more children being diagnosed (15). Additionally, healthcare access has improved, allowing more families to seek and obtain diagnoses for developmental conditions (16). Another contributing factor could be an increase in early screening initiatives and better access to educational resources, which have improved early identification of children with ASD (17). Changes in parental reporting, as well as sociocultural factors such as the reduced stigma surrounding autism, may also play a role in the upward trend in prevalence rates. The difference in autism prevalence between males and females, with boys being diagnosed more frequently than girls, is a well-documented phenomenon in the previous studies (12–14). One reason for this discrepancy may be that autism presents differently in girls, often with less overt or stereotypical behaviors, which can lead to underdiagnosis or misdiagnosis. Girls with autism may also be better at masking or compensating for their symptoms, making it harder for parents and clinicians to recognize their challenges early on.

Differences in the prevalence of developmental disabilities by race, as reported in this study, can be attributed to several factors. Socioeconomic disparities, healthcare access, and cultural attitudes toward disabilities may all contribute to these differences. For instance, non-Hispanic black children exhibited higher prevalence rates for certain developmental disabilities such as ID and LD. This could be due to differences in access to diagnostic and support services (18), particularly in lower-income communities (19), which tend to have a higher proportion of minority populations. Additionally, racial-ethnic minorities often experience systemic barriers in healthcare, such as implicit bias in diagnosis and treatment, which could result in under- or over-diagnosis of certain conditions in different racial groups.

The primary limitation of this study pertains to the assessment of these developmental disabilities including ASD. The data in the NHIS were reported by parents or guardians, and as such, the prevalences were reliant on their reports.

## 5 Conclusions

Our study reveals an increase in ASD prevalence and persistent disparities among racial-ethnic groups. Non-Hispanic black children face elevated risks of ID and LD, while non-Hispanic white children exhibit higher rates of ADHD/ADD. Targeted interventions are essential to address these disparities, emphasizing the importance of inclusive strategies to mitigate the impact of developmental disabilities.

## Data Availability

The original contributions presented in the study are included in the article/supplementary material. Further inquiries can be directed to the corresponding author.
